# Cooperation recruits corticolimbic projections and induces synaptic change in rats

**DOI:** 10.64898/2026.09.07.749944

**Published:** 2026-10-21

**Authors:** Henry W. Kietzman, R. Allie Cauchon, Amelia Johnson, David R. Backer Peral, Robin Bonomi, Yiyun Huang, Hayde Sanchez, Alya Bagdas, Andy Chen, Shreya Saxena, S. William Li, Jane R. Taylor

## Abstract

Cooperation is typically studied as an output of the social brain, as opposed to an experience that shapes it. Here, we developed a task in rats that permits interaction while requiring joint action for reward. Cooperation depended on partner familiarity and visual access. Anterior cingulate cortex (ACC) to basolateral amygdala (BLA) projections were activated during cooperation with a familiar partner, whereas ACC to anterior insula (AI) projections were simultaneously suppressed regardless of familiarity. Using synaptic vesicle glycoprotein 2A (SV2A) positron emission tomography (PET), we found that a single 90 min cooperative experience increased presynaptic density in the amygdala and insula one day later. Cooperative experience enhanced subsequent responding toward a distressed conspecific, in proportion to the amount of cooperation. Cooperation is thus an experience that recruits dissociable corticolimbic pathways and changes brain and behavior beyond the encounter itself.

## Full Text Availability

The license terms selected by the author(s) for this preprint version do not permit archiving in PMC. The full text is available from the preprint server.

